# Polyphenols and metabolism: from present knowledge to future challenges

**DOI:** 10.1007/s13105-024-01046-7

**Published:** 2024-10-08

**Authors:** Sergio Quesada-Vázquez, Itziar Eseberri, Francisco Les, Patricia Pérez-Matute, María Herranz-López, Claude Atgié, Marta Lopez-Yus, Paula Aranaz, José A. Oteo, Xavier Escoté, Silvia Lorente-Cebrian, Enrique Roche, Arnaud Courtois, Víctor López, María Puy Portillo, Fermin I. Milagro, Christian Carpéné

**Affiliations:** 1 Eurecat, Centre Tecnològic de Catalunya, Unitat de Nutrició i Salut, Reus, 43204 Spain; 2 Nutrition and Obesity Group, Department of Nutrition and Food Science, University of the Basque Country (UPV/EHU) and Lucio Lascaray Research Institute, Vitoria, 01006 Spain; 3 Department of Pharmacy, Faculty of Health Sciences, Universidad San Jorge, Zaragoza, 50830 Spain; 4 Instituto Agroalimentario de Aragón-IA2, CITA-Universidad de Zaragoza, Zaragoza, 50013 Spain; 5 Infectious Diseases, Microbiota and Metabolism Unit, CSIC Associated Unit. Center for Biomedical Research of La Rioja (CIBIR), Logroño, 26006 Spain; 6 Institute of Research, Development and Innovation in Healthcare Biotechnology of Elche (IDiBE), Miguel Hernández University (UMH), Elche, 03202 Spain; 7 Equipe ClipIn (Colloïdes pour l’Industrie et la Nutrition), Bordeaux INP, Institut CBMN, UMR 5248, Pessac, 33600 France; 8 Department of Nutrition, Food Sciences and Physiology, Center for Nutrition Research, University of Navarra, Pamplona, 31008 Spain; 9 Navarra Institute for Health Research (IdiSNA), Pamplona, 31008 Spain; 10 Hospital Universitario San Pedro, Logroño, 26006 Spain; 11 Department of Applied Biology-Nutrition, Institute of Bioengineering, Miguel Hernández University (UMH), Elche, 03202 Spain; 12 Alicante Institute for Health and Biomedical Research (ISABIAL), Alicante, 03010 Spain; 13 CIBERobn Fisiopatología de la Obesidad y la Nutrición, Instituto de Salud Carlos III (ISCIII), Madrid, 28029 Spain; 14 Département des Sciences de l’Environnement, Institut des Sciences de la Vigne et du Vin, UMR OEnologie (UMR 1366, INRAE, Bordeaux INP), AXE Molécules à Intérêt Biologique, Bordeaux, 33882 France; 15 Institut des Maladies Métaboliques et Cardiovasculaires, INSERM UMR1297, Toulouse, 31432 France; 16 Team Dinamix, Institute of Metabolic and Cardiovascular Diseases (I2MC), Paul Sabatier University, Toulouse, 31432 France; 17 Present Address: Liver Vascular Biology Research Group, IDIBAPS Biomedical Research Institute, CIBEREHD, University of Barcelona, Spain, 08034 Barcelona, Spain; 18 Adipocyte and Fat Biology Laboratory (AdipoFat), Translational Research Unit, University Hospital Miguel Servet, Zaragoza, Spain; 19 Instituto Aragonés de Ciencias de La Salud (IACS), Zaragoza, Spain; 20 Instituto de Investigación Sanitaria (IIS)-Aragón, Zaragoza, Spain; 21 Department of Pharmacology, Physiology and Legal and Forensic Medicine, Faculty of Health and Sport Science, University of Zaragoza, 50009 Zaragoza, Spain; 22 Aragón Health Research Institute (IIS-Aragon), 50009 Zaragoza, Spain

**Keywords:** Sport, Metabotype, Metabolomics, Inflammation, Prebiotics, Microbiota, Toxicology

## Abstract

A diet rich in polyphenols and other types of phytonutrients can reduce the occurrence of chronic diseases. However, a well-established cause—and—effect association has not been clearly demonstrated and several other issues will need to be fully understood before general recommendations will be carried out In the present review, some of the future challenges that the research on phenolic compounds will have to face in the next years are discussed: toxicological aspects of polyphenols and safety risk assessment; synergistic effects between different polyphenols; metabotype-based nutritional advice based on a differential gut microbial metabolism of polyphenols (precision nutrition); combination of polyphenols with other bioactive compounds; innovative formulations to improve the bioavailability of phenolic compounds; and polyphenols in sports nutrition and recovery.Other aspects related to polyphenol research that will have a boost in the next years are: polyphenol and gut microbiota crosstalk, including prebiotic effects and biotransformation of phenolic compounds into bioactive metabolites by gut microorganisms; molecular docking, molecular dynamics simulation, and quantum and molecular mechanics studies on the protein–polyphenol complexes; and polyphenol-based coating films, nanoparticles, and hydrogels to facilitate the delivery of drugs, nucleic acids and proteins.In summary, this article provides some constructive inspirations for advancing in the research of the applications, risk assessment and metabolic effects of dietary polyphenols in humans.

## Introduction

A myriad of beneficial properties, including antioxidant, anti-inflammatory, antimicrobial, antiproliferative and metabolic effects, have been associated with polyphenol-enriched foods and plant by-products. Consequently, polyphenols appear to be interesting candidates for both pharmaceutical and nutraceutical applications, particularly in the field of metabolic diseases [19].

Many reviews have focused on the beneficial effects of different types of phenolic compounds (flavonoids, stilbenes, anthocyanins, catechins…) or specific molecules (quercetin, resveratrol, epigallocatechin-gallate (EGCG), curcumin, rutin, hesperidin…). Also, huge scientific literature has been built around the effects of this type of molecules on different human diseases and health complications such as obesity, diabetes, non-alcoholic fatty liver (NAFLD), cardiovascular diseases (CVD), and low-grade inflammation.

In this review, we have focused on some of the future challenges that the research on phenolic compounds will have to face in the next years. These points are the following:

- Toxicological aspects of polyphenols and safety risk assessment.
- Synergistic effects between different polyphenols.
- Metabotype-based nutritional advice based on a differential gut microbial metabolism of polyphenols: paving the way to precision nutrition.
- Innovative combinations of polyphenols with other bioactive compounds.
- Innovative formulations to improve the bioavailability of phenolic compounds.
- Polyphenols in sports nutrition and recovery.

Other aspects related to polyphenol research that merit importance are the following:

- Polyphenol and gut microbiota crosstalk: prebiotic effects and biotransformation of phenolic compounds into bioactive metabolites by gut microorganisms [94].
- Molecular docking, density functional theory (DFT) calculations, molecular dynamics simulation, and quantum mechanics/molecular mechanics studies on the protein–polyphenol complexes [1, 87].
- Innovative formulations to increase intestinal absorption and improve the bioavailability of phenolic compounds [120].
- Polyphenol-based coating films, nanoparticles, nanocapsules, and hydrogels to facilitate the delivery of proteins, nucleic acids and drugs [126].

## Consumption of polyphenol-rich foods and safety risk assessment

Currently, the strong recommendations of various national nutrition and health programs emphasize the consumption of many fruits and vegetables. Indeed, their abundance in numerous polyphenols and other types of phytonutrients gives them powerful antioxidant capacities that can potentially prevent or reduce the occurrence of chronic diseases [26, 105]. However, considering the various toxic effects that have been identified for some of these compounds, it would be appropriate to rigorously assess the health risks they may pose in situations of high exposure (such as dietary supplement intake, pharmacological treatments, variations in sensitivity for some populations, etc.). Then, it is necessary to be able to evaluate the risk-benefit ratio associated with the exposure to these multiple molecules of interest in all situations before nutritional recommendations are carried out for the population or certain groups.

For a standard risk assessment procedure, it is important not only to identify the hazard but also to characterize this hazard. While there are several studies dealing with the identification of hazards related to polyphenols (highlighting toxic effects) found in common foods, the characterization of these hazards is often poorly documented. In fact, there are very few comprehensive studies that provide a complete toxicological profile for a specific polyphenol of interest. Those studies are necessary to determine a critical effect and establish toxicological reference values for comparison with population exposure levels or to determine a toxicological benchmark for assessing an exposure margin.

### Polyphenols that induce genotoxic and cancerogenic effects

Caffeic acid is a natural phenolic antioxidant widely distributed in vegetables, fruits and beverages. This polyphenol was shown to act as an antitumor promoter due to its inhibitory effects on mutagenesis and carcinogenesis. However tumorigenic activity of caffeic acid was also suggested since it could induce cell hyperplasia of the forestomach in rats and hamsters. In addition, Hagiwara et al. have observed that the administration of diet containing 2% caffeic acid for 104 weeks in rats and 96 weeks in mice induced the formation of forestomach squamous cell papillomas or carcinomas at high or low incidences in rats and mice, respectively [47, 48]. These authors highlighted tumor lesions in kidneys of both species with higher incidence for male compared to female rats. In addition, caffeic acid exposure also induced alveolar type II tumors in male mice. Even if caffeic acid exposure seemed to be more important than that is encountered in human exposure, those results showed one example of the carcinogenic activity of one polyphenol at least in animal species.

### Polyphenols that disrupt the endocrine system

Some polyphenols are considered phytoestrogens, which correspond to compounds with chemical structures close to human estrogen hormones and, therefore, could perturb endocrine systems [14]. Those effects were well documented in cattle. Polyphenols that belong to the class of isoflavones, and in particular those present in soybeans, such as genistein and daidzein, or in red clover (used to produce dietary supplements) such as formoninetin, are well-known phytoestrogens. Moreover, in the gut, daidzein is converted to equol by intestinal microflora and represents a metabolite with an even closer chemical structure to estradiol and therefore possesses a higher estrogenic activity. Approximately 40 to 70% of adults do not excrete equol to urine, suggesting that only a few proportions of humans could metabolize daidzein to equol (as will be described afterwards) According to in vitro and in vivo studies the estrogenic activity is the highest for estradiol followed by genistein and equol, which are more potent than glycitein, daidzein, formononetin, and biochanin A [62]. Those phytoestrogens are considered agonists at low concentrations and antagonists at high concentrations for the estrogen receptor. Estrogens, and by extension phytoestrogens, are considered to have a positive impact in many physiopathological situations as for example cardiovascular diseases or menopausal symptoms. On the other hand, they may cause harmful effects on the reproductive system and may represent a health concern in women in child-bearing age or in the development of the reproductive system in children. To the best of our knowledge, despite numerous studies have been conducted on the effect of soybean consumption on the sexual development of children, none of them have highlighted such deleterious effects. Nevertheless, animal studies using soybean exposure during pre- and postnatal periods or observations in cattle have revealed negative effects on reproductive systems (signs of estrus in young immature animals, abortion, uterine abnormalities, endometriosis, abnormal development of the mammary glands, disruption of the ovary function). Most of the studies are focused on phytoestrogens, but recent studies have also shown some potential estrogenic effects of stilbenes, particularly for resveratrol [99].

### Polyphenols that affect thyroid function

The thyroid gland produces thyroid hormones, namely triiodothyronine (T3) and thyroxine (T4), that are involved in the metabolism, growth, and development of the human body. Several plant constituents are known to interfere with thyroid hormone synthesis and functions. The decrease in T3 and T4 production led to an increase in thyroid stimulating hormone (TSH) that stimulates the thyrocytes growth and the enlargement of the thyroid gland (goiter). In fact, the effects of plant constituents on thyroid function were highlighted more than 60 years ago in the population of West Africa was and were further attributed to the consummation of millet rich in glycosylflavones [36]. Later, many types of plant constituents including polyphenols were shown to interfere with thyroid hormones synthesis or functions at different levels such as a competition with enzymes involved in the thyroid hormones’ genesis, a down-regulation of the expression of thyroid specific genes, a perturbation of iodide homeostasis, or an induction of thyroid morphological changes. As an example, the flavonol quercetin was shown to inhibit the thyroperoxidase activity and tyrosine iodination (one of the first step for thyroid hormones synthesis), to decrease thyroid specific genes expression in in vitro models and to decrease iodide uptake in vivo in rats [42, 45]. The effects of other polyphenols that belong to the class of flavonoids (flavonols, flavones, isoflavones, flavanones and flavanols) and non-flavonoids (stilbenoids, phenolic acid) have been reviewed by Di Dalmazi and Giuliani [25].

### Prediction of polyphenols toxicity: the Tox DP2 database

In general, natural compounds derived from plants are commonly perceived as safe by the public. Among them, since polyphenols present a growing interest in human health, some are used as dietary supplements and are administered at high doses, and in such cases could induce detrimental effects in consumers. As described in the previous sections, only a few data are available about polyphenols’ acute or chronic toxicity and there is a need to increase our knowledge of the safety of these compounds. In order to overcome this issue, Sinha et al. [115] developed a computational database dedicated to the toxicity prediction of dietary polyphenols, the ToxDP2 database. This database is freely available at the following website: http://ctf.iitrindia.org/toxdpp/. It compiles biological, chemical, and toxicological information for more than four hundred dietary polyphenols categorized into four major classes such as stilbenes, lignans, phenolic acids and flavonoids. Absorption, distribution, metabolism, excretion and some toxicological properties such as organ toxicity, mutagenicity, carcinogenicity, developmental toxicity, and skin sensitization, were obtained using validated toxicological prediction tools (Discovery Studio Program, QSAR). This database represents a unique resource that participates to the understanding of polyphenol structure toxicity relationships and may help manufacturers in the development of nutraceuticals.

### Assessment of the health benefice-risk associated with exposure to three classes of polyphenols

#### Exposure to resveratrol (RSV)

The most well-documented studies rely with the most used polyphenols and those that have been the subject of authorization requests due to their status as new foods or new ingredients intended for commercialization as dietary supplements or for incorporation into food products. To assess the benefice risk ratio associated with polyphenols exposure through food consumption or clinical use, Table 1 presents data on the risk assessment of three classes of polyphenols: resveratrol (RSV), epigallocatechin gallate (EGCG), and curcuma or its active compound, curcumin. Additionally, Table 2 outlines the potential benefits of using these three polyphenols to prevent or alleviate various symptoms associated with human metabolic disorders such as diabetes, cardiovascular functions or lipid metabolism. For RSV, European Food Safety Authority (EFSA) assessed its safety in 2016 [28] as part of a marketing authorization request for its use in dietary supplements, with a maximum recommended daily intake of 150 mg per kilogram of body weight. This report involved compiling various existing toxicology data on RSV and led to the establishment of a toxicological reference point (BMDL) of 344 mg per kilogram of body weight per day based on a chronic study performed in rats [27].

Comparing this value to the typical exposure of people to RSV through regular food consumption (ranging between 0.86 and 2.93 mg*kg^−1^*bw*day^−1^) allowed for the determination of a safety margin of exposure (MOE) ranging between 400 and 177, thus eliminating any safety concerns associated with such high levels of consumption. However, this MOE decreases to 2.3 for individuals exposed to resveratrol through a combination of regular food and dietary supplements, potentially raising safety concerns (see Table 1). Findings from clinical trials highlighting beneficial impacts on various metabolic conditions in humans clearly demonstrate that the maximum effective doses given to patients, 1000 mg*day^−1^ (or 15.5 mg*kg^−1^ bw*day^−1^), for an average human weight of 70 kg (see Table 2), and even doses that trigger adverse effects, are more than twenty times lower than the toxicological reference value identified in animal studies. The average daily exposures to RSV and its derivatives (and metabolites) for a normal diet as reported by a study on a Spanish cohort [133] and other studies on other European cohorts range from 100 to 200 µg/day, considering wine and grape juices as the major source of resveratrol.

Based on a comparison of the maximum doses used to achieve prevention or reduction goals for disorders associated with several metabolic pathologies, with doses established as toxicological reference points derived from animal studies, the use of RSV in humans does not appear to pose a major risk of developing serious adverse effects. However, the calculated safety margins are still relatively narrow, requiring significant monitoring for the emergence of adverse effects.

### Exposure to epigallocatechin gallate (EGCG)

Infusions or preparations based on green tea are generally recognized as not presenting any health risks to consumers [29]. However, a few cases of liver toxicity, which are rather idiosyncratic in nature, have been observed in individuals who consumed tea infusions. Based on these observations, along with clinical study results, EFSA concludes that the risk of increased alanine aminotransferase levels (a marker of hepatic damage) cannot be ruled out for doses of dietary supplements equivalent to more than 800 mg of EGCG per day [29]. EFSA also states that it is not possible to establish a dose of EGCG that can be considered “safe.” Indeed, the dose of 800 mg per day is slightly higher than the exposure levels typically reached by daily tea consumers (between 90 and 300 mg of EGCG per day). However, it does not eliminate the possibility that very heavy tea drinkers in the population may reach these consumption levels, especially considering that the pharmacokinetic of EGCG in the plasma and the toxic effects can be highly dependent on the matrix in which they are contained. Like for RSV, EGCG has been used in clinical studies in humans and has shown promising effects on weight loss, cardiovascular diseases, type 2 diabetes, and complications related to oxidative stress damages (see Table 2). The maximum values used in these studies, 700 mg/day or 10 mg*kg^−1^*day^−1^, are also much lower (about 20 times lower) than the toxicological reference point (NOAEL of 242 mg*kg^−1^ bw*day) identified as the threshold for toxic effects in animals.

### Exposure to curcuma or its active substance: curcumin

Many dietary supplements containing turmeric extracts or its active compound, curcumin, are marketed for their potential protective digestive or hepatic effects, explained by antioxidant, or anti-inflammatory mechanisms. Unlike the other two previously mentioned dietary supplements, an acceptable daily intake (ADI) has been established for curcumin. In 2004, the Joint FAO/WHO Expert Committee on Food Additives [56] set the ADI for curcumin at 3 mg*kg^−^* bw*day^−1^, based on a reduction in body weight gain in the second generation of rats exposed to the highest dose in a developmental toxicity study. The current consumption of dietary supplements containing curcumin, especially those with increased bioavailability (achieved through encapsulation techniques or by the addition of other substances like piperine), can exceed this ADI. Since 2019, several reports from the ANSES nutrivigilance service have identified liver disorders associated with high intake of these dietary supplements [3]. The consumption of curcumin and other derivatives from extracts of turmeric in humans generally ranges from 0.2 to 0.6 g per day, and toxic doses observed are fifty times higher. However, these quantities could potentially be reached with forms that significantly increase bioavailability.

### Assessment of the health conclusion

Bibliographic studies and reports from various assessment agencies do not highlight any health risk of concern to consumers exposed to polyphenols, at least for normal consumption of foods containing these phytonutrients. However, high exposures observed in heavy consumers or consumers of highly dosed dietary supplements or in formulations that modified the bioavailability, do not rule out the risk of adverse effects, as mentioned in animal studies or intervention clinical studies in humans. So, such situations of increasing consumption of dietary supplements accompanied by significant changes in dietary behaviors promoting the consumption of plants could quickly lead to safety concerns. It is therefore urgent to conduct appropriate studies to establish toxicological profiles of the most commonly used polyphenols, especially for those used as dietary supplements, in order to proceed to robust heath safety evaluations.

**Table 1:** Synthesis of the safety risk assessment of consumers exposed to three classes of polyphenols (stilbenes: resveratrol - flavanols: EGCC - Curcuminoids: curcumin) via global food consumption or global food plus food supplement consumption

Classes of Polyphenols (type of polyphenols)	Toxicological adverse effects	Reference value (POD or VTR) mg*kg^-1^ bw*d^-1^	Normal exposure (NE) mg*kg^-1^ bw*d^-1^	High exposure (HE) mg*kg^-1^ bw*d^-1^	Risk characterization MOE or % of DJA	Risk evaluation	Ref.
Stilbenes (Resveratrol)	Decrease of body weight	344 (BMDL)	0.86–2.93	150	172 (NE) < 100 (HE)	No safety concern Safety concerns	[28, 85]
Flavanols (EGCC)	Hepatotoxicity	242 (NOAEL)	1.14–4.28	800	> 100 (NE) < 100 (HE)	No safety concern Safety concerns	[29]
Curcuminoids (Curcumin)	Hepatotoxicity	3 (ADI)	2.85–4.28	1000–1500	> 100% of DJA (NE and HE)	Safety concerns	[3]

**NE** : normal exposition (exposition calculated with data of normal food consumption) – POD (Point of Departure) – VTR (Toxicological Reference Value) - **HE** : High exposition (exposition calculated with data of normal food consumption + food supplement consumption) - BMDL (Benchmark Dose Limit) - NOAEL (Non-Observed Adverse Effect Level) – ADI (Acceptable Daily Intake) – MOE (Margin of Exposure)

**Table 2:** Metabolic beneficial effects of three classes of polyphenols (stilbenes: resveratrol - flavanols: EGCC - Curcuminoids: curcumin) used in clinical studies (interval of doses for maximal efficiency and appearance of secondary adverse effects)

Classes of Polyphenols (type of polyphenols)	Main beneficial effects from clinical studies	Dose: mg*day^-1^) (Treatment duration: day)	Adverse effects from clinical studies	Dose: mg*d^-1^) (Treatment duration: day)	Ref.
Stilbenes (RSV)	**T2DM**: Decrease fasting blood glucose, HbA1c and insulin resistance **Metabolic syndrome**: Decrease of BMI and insulin secretion **Inflammatory diseases**: Reduce CRP, IL-6, and TNF-α plasma concentrations **Myocardial infarction**: Improve left ventricular diastolic and endothelial functions Decrease blood LDL levels	480–**1000** (28–45) 1 500 (90) 40–250 (42–90) 10 (90)	*Digestive symptoms*: Nausea, diarrhea, abdominal pain No side effect was noticed below 1 g/d	2 500-**5 000** (29) 1 000 (8) 4 000 (8)	28 114
Flavanols (EGCC)	**Weight loss**: Increase fat oxidation Decrease of BMI, waist circumference, body fat mass and subcutaneous abdominal fat **Cardiovascular diseases**: Inverse correlation with ischemic heart disease mortality Decrease blood LDL levels **T2DM**: Decrease in HbA1c **Oxidative damage**: decrease in urinary 8-OHdG	570–690 (56–72) Daily consumption above 70 **856** (112) 294 (120)	*Hepatic disease*: Increase in transaminases levels	800-**4 000** (pure or in green tea extracts) (10–360)	29
Curcuminoids (Curcumin**)**	**Lipid metabolism**: Decrease of serum lipid peroxides and total cholesterol Increase in HDL cholesterol **Choleretic properties**: Decrease in bladder volume	**500** (7) 20 (1)	*General symptoms*: Headache, dizziness, nausea, diarrhea *Hepatic diseases*: Cytolytic, auto-immune hepatitis	105-**1 000** 42–90	3

T2DM: Type 2 Diabetes Mellitus – RSV : Resveratrol – EGCG : epigallocatechin gallate

## Synergistic effects between different polyphenols

The combination of active biomolecules can be an interesting tool to prevent or ameliorate several diseases, due to potential additive or synergistic effects. Indeed, combinations could enhance a beneficial effect, reduce the dosage of each compound without compromising the final efficiency and reduce the side effects. This is the case of polyphenol combinations [38]. In the following lines some examples, both in *in vitro and in vivo* models, of the positive effects of polyphenol combinations on inflammation, a process underlying a great number of common diseases in Western societies, adipocyte metabolism and other aspects related to prevalent diseases, such as cardiovascular diseases, are described.

Concerning inflammation, several in vitro studies have analysed the effects of polyphenol combinations on numerous markers in macrophage cellular models, mostly in RAW 264.7 cell line [79]. These studies include combinations of flavonoids (flavanols, flavone, flavanone, isoflavone, flavone-3-ols), non-flavonoids (hydroxycinnamic acid and stilbenes), compounds derived from phenol, and saponins. In most of them the inflammatory markers measured were nitric oxide (NO), prostaglandin E2 (PGE2), interleukins (IL-1, IL-6), tumor necrosis factor α (TNF-α), inducible NO synthase (iNOS), and cyclooxygenase (COX). Interestingly, in some studies reactive oxygen species (ROS) were also measured.

Most of the results indicate that the combinatory effects were either additive or synergic, at low doses, whereas they were antagonist at high doses. As an example, Murakami et al. measured the inhibition of NO production in lipopolysaccharide (LPS)-stimulated RAW 264.7 cells, and demonstrated that the interactive effect was different depending on the concentration of given compounds. Synergism occurred only when epigallocatechin gallate (EGCG) was combined with genistein (GEN) at low concentrations (EGCG at 0.04 µM and GEN at 2 µM), whereas at higher concentrations (EGCG at 10 µM and GEN at 25 µM) the effect was antagonistic. In addition, the combination of resveratrol (RSV) and GEN resulted in an additive effect at low doses (RSV at 2 µM and GEN at 2 µM), and an antagonistic effect at high doses (RSV at 50 µM and GEN at 25 µM) [82].

To illustrate the diversity of the compounds’ combinations and their consequences on inflammatory markers, Harasstani et al.. highlighted that several flavonoids as flavanol (kaempferol and morin), flavone (quercetin, chrysin and disomin), and flavanone (silibinin and hesperidin) were able to inhibit the production of NO, PGE2 and TNF-α in LPS-stimulated macrophages to different extents [49]. By comparing their individual effects with those of their combinations, they found either additive or synergic effects. The most potent synergic effect was observed when kaempferol was used in combination with chrysin [49]. Liu et al. realized several mixtures including flavonoids (genistein, naringenin, hesperitin and biochanin A), gingerols (6-gingerol, 8-gingerol and 6-shogaol) and saponins (protogarcillin, pseudoprotodioscin and dioscin), and measured their inhibitory effects on NO secretion in LPS-stimulated RAW 264.7 cells [70]. They found either no interaction, additive or synergic effects, between the gingerol et saponin groups, and between the flavonoid and the saponin groups, depending on the concentration used, but a synergism between the flavonoids and the gingerol groups, and in particular for the combination of biochanin A and 6-gingerol. This synergic effect was also greatly enhanced at lower concentrations and was also observed on the inhibition of the secretion of TNF-α, IL-1 and IL-6 [70].

In another study, the authors used a combination of two flavones, namely luteolin (LUT) and tangeretin (TAN) that differ in the presence of methoxy groups in the latter. They found that these compounds in combination elicited a stronger inhibition of the production of NO, PGE2, IL-1*β*, and IL-6 than the individual compounds alone. Interestingly, these results were confirmed by a more pronounced inhibition of the expression of iNOS and COX when TAN and LUT were used in combination, which suggests that these compounds may act on the signalling pathways involved in the secretion of those inflammatory markers [34].

Altogether, those observations suggest that the nature of the interactive effects depends on the compounds used in the combination and their respective concentrations in the mixture. To explain these observations, some authors suggested that combinations at high concentrations could lead to saturation at the target site related to the inhibitory effects and/or to the emergence of undetectable cytotoxicity [82].

As an obvious link exists between inflammation and oxidative stress, some authors measured the combinatory effects of polyphenols on these two phenomena. A strong synergistic effect was observed regarding the inhibition of NO, TNF, and IL-6, but also on ROS production and COX-2 expression, upon exposure to silibinin and thymol in LPS-stimulated RAW 264.7 cells [15]. In another study, the combination of chicoric acid (CA) and acetoxy chavicol acetate (ACA) resulted in an additive effect at high concentrations or a synergistic effect at low concentrations in the suppression of TPA-induced generation of O_2_^•−^ in HL-60 cells. They also showed that the combinations of EGCG/ACA and EGCG/CA mainly resulted in an antagonistic effect [82]. Once again, these data highlighted that the final biological effects of the combinations depend on the specific compounds and their respective concentrations in the mixture, as it was described before for the anti-inflammatory response. By contrast, LUT and CA both individually suppressed oxidative stress in LPS-stimulated RAW 264.7, but their combination did not result in any synergistic effects on the inhibition of ROS production at any of the concentrations tested, whereas it was able to synergistically inhibit the production of NO and PGE2 [88]. Altogether, despite the well-documented relation between inflammatory response and oxidative stress, some combinations could synergistically inhibit inflammatory response without having any effect on oxidative stress, whereas they individually suppress both responses. Then, the combinatory effects involved complex mechanisms of action that are probably the foundation of the synergism.

The signalling pathways involved in the combinatory effects of polyphenols were assessed by several research groups. During the inflammatory response, the translocation of NFkB into the nucleus is an important step for its activation, which further can lead to the stimulation of the expression of inflammatory genes, resulting in the secretion of inflammatory markers. This occurred through the phosphorylation of Iκβ that releases NFκβ to allow its translocation into the nucleus and the stimulation of inflammatory gene expression. In this context, the combination of LUT and CA was shown to suppress the phosphorylation of Iκβ. Authors also showed that the combination of LUT and CA could inhibit Akt phosphorylation, but had no effect on the phosphorylation of MAPK (p-p38, p-ERK and p-JNK) [88]. To our point of view, this represents an interesting observation, since it was shown that the combination of other polyphenols such as quercetin (QUER) and catechin (CAT) or the combination of silibinin and thymol could also inhibit the activation of NFκβ signalling pathway, but through a synergic inhibition of the phosphorylation of MAPK and therefore their activation [15, 66]. Upon LPS exposure, the expression of TLR4 and MyD88, both factors involved in the first step of the NFκβ signalling pathway, was enhanced and the combination of QUER and CAT could restore to a basal level the expression of those factors, especially for MyD88, in a synergistic manner [66]. These observations highlight the fact that even if the NFκβ signalling pathway seemed to be the common target of polyphenols to suppress the inflammatory response, its inactivation results from the action of polyphenols combination at different levels of its signalling pathway. Another hypothesis could reside in the inhibition of different signalling pathways that depends on the nature of the polyphenols present in the mixture. Therefore, if a combination with polyphenols acting on different signalling pathways is found, the final biological effects could be improved either to a greater extent or to the same extent but with lower doses of polyphenols.

The effects of polyphenol combination on inflammation and oxidative stress have also been studied in the frame of some diseases by several authors. Thus Liu et al. analysed the implication of NFκβ pathway in the effectiveness of the plant extract of *Polygonum cuspidatum* Siebold & Zucc. as a therapeutic agent in an ulcerative colitis mouse model [68]. Resveratrol and polydatin were described as the main phenolic compounds present in the extract, and the authors analysed their individual and combined effect (with emodin, the next most present component in the extract) on the NFκβ pathway signalling in mouse intestinal cells ex vivo. The presence of some proteins, such as TNFα, interleukins and Bcl2 was analysed in the incubation media of cells, and an inhibitory effect of the combination of both compounds was observed to be higher than that of the individual ones.

In 2019, Liu et al. studied the combined effect of eriocitrin and resveratrol as anti-inflammatory agents in lipopolysaccharide (LPS)-induced RAW264.7 cells and a mouse model of ear oedema [69]. The combination of eriocitrin and resveratrol showed a stronger inhibitory effect on the production of nitric oxide induced by LPS in RAW264.7 macrophages; in fact, the reduction after the combined treatment was more than the sum of both individual treatments, suggesting an additive effect. Moreover, the authors measured the protein expression of some pro-inflammatory and anti-inflammatory cytokines and proteins involved in MAPK and NF-κB pathways, and they concluded that the mechanism of action for the additive effect involves multiple pathways, as previously described in this section. Concerning the in vivo experiment, the authors observed that both compounds in combination were much more effective in reducing ear swelling in animals than the individual ones, which had little effect [69].

Other authors have analysed the effects of polyphenol combinations on oxidative stress related to parameters related to cardiovascular diseases. Thus, Vivancos et al. [124]. analysed the individual and combined effect of two doses of resveratrol (3 and 30 µM) and tyrosol (10 and 100 µM) on the oxidised LDL-induced H_2_O_2_ production in RAW 264.7 macrophages. Interestingly, they observed that whereas at the highest dose both molecules were effective either alone or in combination, at the lowest dose only the combination reverted the H_2_O_2_ production induced by oxidised LDL.

To finish this section, the effects of polyphenol combinations on adipose tissue alterations are presented. In a study performed by Vazquez-Prieto et al. [123], the authors aimed to evaluate the effectiveness of quercetin, catechin or their combination on the amelioration of adipose inflammation both in in vitro and in vivo conditions. The authors analysed the effect of 1 and 10 µM of catechin, quercetin or their combination in the reduction in adiponectin secretion induced by TNFα in 3T3-L1 adipocytes. Although all the treatments restored adiponectin secretion, the effect was stronger with the combination at both doses, specially comparing them with the individual treatment at 1 µM. Furthermore, rats fed a high fructose diet developed dyslipidaemia and showed a reduction in plasma adiponectin, adiposity, adipose tissue inflammation and insulin resistance, parameters that were reverted by the supplementation. However, whereas in some cases the positive effects were similar in animals supplemented with catechin, quercetin or the combination, in others the combination was more effective. For example, the MCP-1 increase induced by the high fructose diet was reverted by catechin supplementation, but the combination did not exert a stronger effect. By contrast, the combination of catechin and quercetin was more effective in reducing serum triglycerides and TBARS than the individual supplementation.

Concerning the potential anti-obesity action of polyphenol combinations, several studies have been reported addressing the effects of resveratrol + quercetin under in vitro and in vivo conditions. Yang et al. [131]. showed that a combination of resveratrol (25 µM) and quercetin (25 µM) suppressed lipid accumulation more than the responses observed with resveratrol or quercetin alone, and more than the calculated additive response of both phenolic compounds in 3T3-L1 pre-adipocytes and mature adipocytes. In the case of pre-adipocytes, the delipidating action was due to the inhibition of adipogenesis. Regarding mature adipocytes, the treatment with resveratrol and quercetin, at concentrations of 50 or 100 µM, resulted in an increase in apoptosis, compared to the predicted additive response. The same group reported similar results by combining quercetin, resveratrol and genistein [89]. In this study, when quercetin (12.5 µM), resveratrol (12.5 µM) and genistein (6.25 µM) were combined, lipid accumulation in 3T3-L1 pre-adipocytes was almost completely inhibited, showing an effect clearly higher than the calculated additive individual effect of each compound.

These studies provided interesting and promising results, which need to be checked under in vivo conditions, because the potential interactions in absorption and metabolism among biomolecules cannot be studied in cultured cells. In this regard, it should be remembered that resveratrol shows a low bioavailability, due in part to the strong phase II metabolism that it suffers, both in the gut and the liver. Consequently, after oral administration, the concentration of resveratrol in serum is clearly lower than those of its glucuronide and sulphated metabolites. De Santi et al. [21]. demonstrated, by carrying out in vitro studies, that quercetin was a potent inhibitor of resveratrol sulphation, and, therefore, it could increase resveratrol bioavailability. These results were supported by early studies showing that wine inhibited phenol and catechol sulphotransferases [40, 67].

Taking this in mind, Arias et al. [4]. addressed a study with the following experimental groups of rats: (a) the group fed a high-fat diet, (b) the group fed a high-fat diet treated with resveratrol (15 mg/kg body weight/d), (c) the group fed a high-fat diet treated with quercetin (30 mg/kg body weight/d), (d) the group fed a high-fat diet treated with resveratrol (15 mg/kg body weight/day) and quercetin (30 mg/kg body weight/day). After 6 weeks of treatment, the administration of either resveratrol or quercetin separately did not induce significant reductions in adipose tissue weights. By contrast, the combination of both molecules led to a significant reduction in all the fat depots analysed. The percentage of reduction in each tissue was greater than the calculated additive effect, demonstrating a synergistic effect between resveratrol and quercetin, which is in good accordance with a potentially higher bioavailability of resveratrol in the rats supplemented with both polyphenols than in those supplemented with resveratrol only. In this group of rats, browning of perirenal white adipose tissue was also observed in those treated with the combination of both phenolic compounds, but not in those receiving either resveratrol or quercetin individually [5].

Summing up, despite the evidence of the interaction of polyphenols in biological responses, some questions remained to explain their combinatory effects. For instance, do the polyphenols in combination stimulate or inhibit a common target in a more intense manner or do they act on different targets which result in an enhancement of the biological responses? Also, could they interact with each other to form a new ligand with an enhanced efficacy or selectivity?

It should be pointed out that most of the in vitro results have been obtained with pure compounds, and the combinatory effects that could reside within the uses of plant extracts are scarce and deserve further investigation. The interest in the use of combination also resides in the low doses of polyphenols to reach the biological effects in in vitro studies. In line with this, most of the studies dealing with the pharmacokinetics of polyphenols in animals have reported low blood concentrations. Despite their low blood concentrations, when they are in combination in a plant extract for example, polyphenols could exhibit some interesting biological effects as described in the former paragraphs. The effects of these polyphenols in humans, both in combination or as part of foods, deserve further investigation too.

## Synergistic and additive effects of the combination of polyphenols with nutraceuticals

The Mediterranean diet is a dietary pattern rich in bioactive compounds that inspire the formulation of multi-ingredient supplements based on the beneficial effects of this diet [116]. Therefore, strategies based on the synergistic combination of bioactive compounds to fight the different factors that influence the onset and evolution of these pathologies are the main objective to find the perfect combination between polyphenols and the treatments used in this study. Many of the health problems of metabolic diseases have a multifactorial origin [44]. In this context, the possibility of using different combinations of polyphenols or specific combinations of polyphenols with other nutraceuticals appears with the aim of increasing (synergistically or additively) the beneficial effects of these compounds individually. Polyphenols have been classified depending on their chemical structure, such as focusing on the number of phenol rings contained therein and the structural components connecting these rings, or on their beneficial activity, such as anti-inflammatory, anticancer, antidiabetic, and antioxidant activities [79]. Thus, with the aim of using polyphenols as treatments for metabolic disorders, synergistic or additive combinations of polyphenols have been proposed in several studies due to the multifactorial nature of these metabolic diseases. However, a small number of polyphenols were observed to provide synergistic beneficial effects when administered in combination [79]. Therefore, the need to find new combinations between polyphenols and other bioactive compounds in order to find synergistic effects that can ameliorate affected metabolic pathways led to the focus on different nutraceuticals as possible candidates.

Nutraceuticals provide medical or health benefits, including the prevention and/or treatment of a disease, and protection of the human body against harmful metabolic changes [58]. On the other hand, functional food is consumed as a part of a diet that has a physiological benefit to human health and reduces the risk of suffering from chronic diseases [111]. Therefore, nutraceuticals and functional food, such as metabolic cofactors or organic molecules, have been considered promising alternatives with the ability to induce beneficial effects in the cellular mechanisms and biochemical pathways disrupted or affected from the onset of the metabolic diseases until their progression. Besides, metabolic disorders are multifactorial diseases in which mechanisms are associated with fat accumulation, insulin resistance, inflammation, oxidative stress, mitochondrial dysfunction, endoplasmic reticulum stress, bacterial overgrowth, and genetic predisposition [6]. Hence, this evidence supports the use of a combination of bioactive compounds as a promising strategy to tackle multifactorial metabolic disorders. A clear example of nutraceuticals that can activate or recover altered metabolic pathways is the supplementation of metabolic cofactors, which could be used as a new therapeutic approach against obesity and NAFLD pathogenesis by improving metabolic parameters in the diseases and stopping NAFLD progression to severe stages of this disease including NASH, cirrhosis, and hepatocarcinoma [74, 102]. Therefore, the combination of different nutraceuticals and functional food is a promising strategy to tackle the different hits implicated in the pathogenesis of metabolic disorders.

Metabolic cofactors have been demonstrated to have antilipemic, anti-inflammatory, antifibrotic, and antioxidant properties in NAFLD, obesity-induced disrupted adipose tissue and intestinal dysfunction [100, 101]. In this context, the supplementation with a combination of metabolic cofactors (L-carnitine (LC), nicotinamide riboside (NR), N-acetyl cysteine (NAC), and serine or betaine) was used as a strategy to treat NAFLD because these metabolic cofactors were elucidated to participate in the modulation of affected metabolic pathways in this hepatic disease [75], and, thereby, this combination was proposed to be used as treatment against multifactorial metabolic disorders. _To boost these potential characteristics, polyphenols are an interesting option due to their ameliorative properties against metabolic disorders observed in different studies [2, 71]. Previous studies showed that combinatorial strategies using polyphenols can be an effective strategy against different diseases by synergistically or additively enhancing the efficacy of their beneficial properties [76, 113], and multidisciplinary efforts are necessary to determine the most favorable combinatorial strategies.

Therefore, it is tempting to propose the formulation of a potential combination of specific polyphenols and/or nutraceuticals that may provide additive or synergistic effects against different therapeutic targets increasing health benefits significantly [38]. These innovative combinations will enhance the antilipidemic, antioxidant, and anti-inflammatory properties of these compounds, ameliorating affected metabolic pathways. Indeed, there are several previous studies where the combination of nutraceuticals conferred synergistic and additive effects on different health problems, such as cardiometabolic diseases or cancer [64, 84, 110, 119]. However, the combination of metabolic cofactors with polyphenols is poorly studied, although separately these nutraceuticals have been observed to be promising compounds with widely described benefits on health [63, 80]. Thus, this section will hypothesize that the combination of different functional metabolic cofactors with polyphenols will boost the beneficial health effects of these metabolic compounds and may be used in a mixture as novel treatments to reverse obesity and NAFLD progression through the amelioration of adipose tissue dysfunction and hepatic steatosis. This amelioration should activate anti-inflammatory processes within the hepatic, adipose, and intestinal tissue and promote gut microbiota homeostasis through the synergistic and additive effects of the newest combination.

Synergic effects are strongly dependent on the bioaccessibility and bioavailability of the substances, which are susceptible to be changed by many different factors including food processing, chemical properties of the bioactive compounds, and modifications that occur during digestion [92, 122]. The biological activities of bioactive compounds are dependent on their bioaccessibility and bioavailability which may be modified by the presence of another nutraceutical or organic component [93]. However, polyphenols have an advantage because they are the largest group of nutraceuticals studied for their chemo-preventive effects tested in several studies [122]. One of the main characteristics of polyphenols is their bioavailability and metabolic fate when they are processed and digested in the gut [39]. Most of the polyphenols are hydrolyzed by intestinal enzymes or by bacterial degradation in the large intestine, and polyphenols showed higher biological activity than individual ones [134]. Therefore, the synergistic characteristic of polyphenols is the most important feature that makes them the best option to find synergistic activity with other bioactive compounds.

Considering the beneficial properties of the different polyphenols and metabolic cofactors, the combination of NAC and betaine with antioxidant polyphenols like green tea extracts, anthocyanins, carotenoids, and flavonoids may improve the antioxidant capacity of these compounds, indicating potential synergy [122]. Regarding resveratrol and quercetin, it was numerously used in combination showing a restorative effect on disturbed energy metabolism, lipid accumulation, fatty acid oxidation, and inflammatory responses both in adipocytes and in the liver, which presents both polyphenols as promising compounds to be combined with metabolic cofactors to improve the beneficial effects of the treatment on metabolic disorders [113]. Moreover, carotenoids were also correlated with anti-inflammatory properties in combination with other compounds [46].

Previous studies have demonstrated an ameliorative effect combining polyphenols with the selected metabolic cofactors used in studies mentioned above, such as NAC, NR, LC and betaine, and these studies are specified in Table 3. As relevant studies, we found that NAC mixed with a combination of polyphenols was useful in modulating immune system function implicated in cellular carcinogen mechanisms and decreases oxidative stress associated with anti-fibrotic potential [108, 109]. Moreover, the combination of LC with polyphenols promoted a lipid-lowering effect in hepatocytes by increasing FFA oxidation improving pathological changes in the liver and ameliorating obesity and WAT weights [104], and doses based on the combination of nicotinamide riboside and pterostilbene displayed increased circulant NAD^+^ levels in humans, lowering markers of hepatic inflammation in patients with NAFLD [22]. Therefore, the synergistic combination of polyphenols with the combination of these metabolic cofactors may improve the beneficial effects of these treatments on cardiometabolic diseases and other health problems.

Finally, data from previous studies suggest that the additive or synergistic combination of polyphenols with metabolic cofactors could improve protection against multifactorial pathologies by consistent amelioration of lipid accumulation, oxidative stress, and inflammation. However, further new and well-designed studies are necessary to decipher the relationship between specific polyphenols and detailed metabolic cofactors, which will help us gain novel insight into developing new therapeutic options for metabolic disease prevention and treatment.

**Table 3:** Synergistic combinations between metabolic cofactors and polyphenolic compounds

Synergistic interactions	Doses	Subjects	Effects	References
Nicotinamide riboside (NR) + Pterostilbene (PT)	NRPT at the recommended dose (NRPT 1X; 250 mg of NR plus 50 mg of PT), and NRPT at a double dose (NRPT 2X; 500 mg of NR plus 100 mg of PT)	120 participants in a randomized, double-blind, placebo-controlled repeat dose clinical trial.	Increases NAD + levels in humans	23
Nicotinamide riboside (NR) + pterostilbene (PT) + silibinin (SIL) + fibroblast-stimulating lipoprotein 1 (FSL1)	NR oral administration (185 mg/kg/ day) + 100 mg PT/kg and 100 mg SIL/kg	Swiss albino mice. Mice received c-radiation (137Cs) (total body irradiation, TBI.	The combination of the radioprotectors PT and SIL with the radiomitigators NR and FSL1 confer effective, long-term protection against c radiation	86
Nicotinamide riboside (NR) + pterostilbene (PT)	(1) four placebo capsules, (2) recommended dose: 250 mg NR and 50 mg PT (two NRPT capsules and two placebo capsules), or (3) double dose: 500 mg NR and 100 mg PT (four NRPT capsules).	A randomized, double-blind, placebo-controlled trial with healthy adult males and females, aged 18–70 years, and overweight or obese (body mass index [BMI], 25.0–39.9 kg/m2) and had HFF ≥ 15% by MRI-PDFF.	NR + PT at the recommended dose is safe and may hold promise in lowering markers of hepatic inflammation in patients with NAFLD.	22
Nicotinamide mononucleotide (NMN) + resveratrol or ginsenoside Rg3&Rh2	NMN (50 mg/ml), resveratrol (5 mg/ml), and Rg3&Rh2 (5 mg/ml) were individually suspended in phosphate-buffered saline (PBS) solution for oral study.	Male C57/BL6 mice at 6–8 weeks of age	NMN combined with resveratrol could increase the levels of NAD+ in the heart and muscle, and NMN coupled with ginsenoside Rh2&Rg3 could effectively improve the level of NAD+ in lung tissue	7
L-carnitine (LC) + resveratrol + isoflavone	LC (400 or 800 mg/kg) daily by oral gavage for about 8 weeks. The ratio of resveratrol, soy isoflavone and LC was determined to be 4:1:2.5 by weight.	Specific pathogen-free 7-week-old male C57BL/6 mice were fed with a high-fat diet for 3 weeks before sample treatment	LC + polyphenols suppressed HFD-induced obesity and suggest that LC + polyphenols supplementation might be a promising adjuvant therapy for the treatment of obesity and its complications	59
L-carnitine (LC) + grapeseed extract + others	L -carnitine (1 g), grapeseed extract (81 mg)	Forty-two individuals (13 females, 29 males) with proven hyperlipidemia	Reduction of plasma lipids in hyperlipidemic subjects	104
L-carnitine (LC) + *Prumus* mume stem barks extract (PMSBE)	PMSBE and L-carnitine in the proportion of 2:1 (w/w)	Fifty 7-week-old male ICR HFD-induced obese mice	LC + PMSBE have significantly anti-obesity and hypolipidemic effects and improved the pathological changes in liver tissue and decreased the relative weights of epididymal and perirenal WAT.	130
A grape extract–green tea catechin–L-carnitine (R is resveratrol, GT is green tea and LC is L-carnitine) combination mixture	The combination mixtures (300 mg/kg or 1200 mg/kg) were given daily by oral gavage for about 8 weeks and were prepared by mixing R, GT, and LC and the mixing ratio was 1: 0.6: 0.5 by weight.	Specific pathogen-free 7‐week‐old male C57BL/6 mice fed with HFD for 3 weeks before sample treatment.	LC + GT + R suppressed HFD-induced obesity, hyperlipidemia, and non-alcoholic fatty liver disease	59
L-carnitine (LC), green tea extract (GT) and lotus leaf extract (LL)	Each tablet (717.06 mg/tablet) of compound plant-based supplements contains L-C, GT (80 to 120 mg of catechin per tablet), and LL	60 male Sprague Dawley rats aged 6 weeks were fed with a high-energy diet for 5 weeks.	This combination significantly reduces final body weight, body fat amount, body fat ratio, feed efficiency, and calorie efficiency	128
Curcumin + N-acetyl cysteine (NAC)	Curcumin treatment from 20 µM to 40 µM for induction of antifibrotic effects and the addition of 10 mM NAC	Primary epithelial cells and fibroblasts isolated from patients with Idiopathic Pulmonary Fibrosis	Co-administration of curcumin and NAC decreases oxidative stress, maintains high cell viability, and maintains an anti-fibrotic potential.	104
N-acetyl cysteine (NAC) + quercetin and green tea extract + others	N-acetyl cysteine (200 mg), standardized green tea extract (80% polyphenol, 1000 mg); quercetin as quercetin dihydrate (50 mg)	Athymic female mice inoculated subcutaneously with 3 × 10^6^ ovarian ES-2 cells	Tumor weight was reduced by 59.2% (*p* < 0.0001) and tumor burden by 59.7% *p* < 0.0001)	109
Aleurone (phenolic compounds + Betaine)	40–50 g/day in the Aleurone diet	Overweight/obese subjects of both sexes, with ages between 20 and 70 years. Randomized sequential cross-over design	An 8-week Wheat Aleurone Diet improves the oxidative stress and increases plasma betaine levels in overweight/obese individuals with an increased cardiometabolic risk	20

## Metabotype-based nutritional advice based on a differential gut microbial metabolism of polyphenols: paving the way to precision nutrition

Most systematic reviews and meta-analyses focused on the effects of polyphenols on health underline the promising and potential beneficial effects of these phytochemicals against several high-impact diseases, as suggested before [52, 54, 119]. However, a well-established cause—and—effect association has not been clearly demonstrated because of the heterogeneous, inconclusive, and even controversial results observed. Understanding why some bioactive plant compounds work effectively in some individuals but not, or with a smaller magnitude in others, is crucial for using these natural compounds in future strategies of personalized nutrition and/or for the development of food products for specific population groups [41].

 The study of the inter-individual variation in responsiveness for dietary plant bioactive compounds is a very active research field (proof of that is the COST Action POSITIVe) [41]. Age, sex, ethnicity, physiological status (body mass index-BMI), lifestyle (physical activity and dietary habits), medication, genetic background and the heterogeneity in the methodological approaches carried out could be some of the factors responsible for the huge variability observed in humans [18, 41]. Moreover, in most of the studies it is not clear whether the effects after polyphenol-rich food consumption are mainly exerted by: (i) the ingested phenolic compounds themselves, (ii) their phase-II derived metabolites, (iii) the greater or lesser amount of phenolic-derived metabolites produced by the microbiota (metabolite «gradient», quantitative criterion), and/or (iv) the specific gut microbiota associated with the metabolism of such polyphenols, known as «gut microbiota metabotypes», the key point of this Sect. [54]. In this context, and following the well-known two-way interaction between polyphenols and gut microbiota [18], variations in the gut microbiota composition and functionality among individuals could affect polyphenol metabolism capacity and could explain, at least in part, the large inter-individual differences observed in the production of such bioactive polyphenol-derived metabolites (postbiotics). However, at the same time, polyphenol-related metabotypes can indirectly reflect the individuals’ gut microbiome and the individuals’ health status. Thus, they could be used as «pre-treatment gut biomarkers» to predict the response to specific dietary interventions in the context of personalized nutrition, optimising and aiming the diet individually for the prevention of some diseases. This is a research field of great interest nowadays.

But, at this point, what in essence is a “metabotype”? This term was initially coined by Bolca et al. [9] as an extensive concept involving individuals’ differential metabolic responses to nutritional or pharmacological interventions. Specifically, in the context of polyphenols’ metabolism and health, metabotype refers to a differential metabolic phenotype defined by specific metabolites derived from the gut microbiota, characteristic of the polyphenol precursor ingested and not present in other individuals [30]. In addition, a metabotype is also defined by a characteristic microbial profile in terms of composition and functionality/activity. Therefore, this concept refers to a «qualitative» criterion (producer vs. non-producer) and is not, or minimally influenced, by external factors (diet, motility of gastrointestinal tract, food matrix, sample collection time, etc.).

The metabolism of many polyphenols, including flavanones, lignans, prenylflavonoids, proanthocyanidins, anthocyanins and stilbenes, shows high interindividual variability. However, no gut microbiota-associated metabotypes have been described for them since a metabolite production gradient has been reported giving rise to the so-called “high producers” and “low producers” of certain metabolites rather than a simple classification into responders/non-responders [54]. In fact, the only proven metabotypes described up to now, following the aforementioned definition, are those involved in the metabolism of isoflavones-daidzein (equol and/or O-desmethylangolesin producers vs. non-producers), and ellagic acid (urolithin metabotypes (UMs): UM-A: producers of only Uro-A; UM-B: producers of Uro-A, isourolithin-A (IsoUro-A), and urolithin-B (Uro-B); and UM-0 (urolithin non-producers) [reviewed in 18 and 54].

All these metabotypes (both equol and urilithin-derived metabotypes) showed a distinct and characteristic microbiome profile. Thus, more than 10 gut microbes seem to be involved in the equol production, including *Adlercreutzia equolifaciens*, *Adlercreutzia equolifaciens subsp. celatus*, *Bacteroides ovatus*, *Bifidobacterium bifidum*, *Finegoldia magna*, *Limosilactobacillus mucosae*, *Slackia equolifaciens*, *Slackia isoflavoniconvertens* and *Streptococcus intermedius* [55]. In addition, the equol-producer metabotype showed a higher abundance of *Prevotella*, *Megamonas*, *Allistipes*, *Desulfovibrio*, *Collinsella*, and *Eubacterium* genera. In contrast, the equol non-producer metabotype was enriched in the family Lachnospiraceae, the genus *Eggerthella* and several species from *Ruminococcus* and *Bacteroides* [136]. No differences were observed in terms of microbial diversity/richness among equol producers and non-producers. In contrast, the UM-0 metabotype showed lower diversity and microbial richness than the UM-A and UM-B metabotypes. Besides, UM-0 was characterised by a lower abundance of the genera *Phascolarctobacterium*, *Bilophila*, *Alistipes*, and *Butyricimonas* compared to UM-B and UM-A. *Gordonibacter* species were positively associated with Uro-A and UM-A in faeces and urine whereas UM-B showed a higher abundance of Coriobacteriia and *Ellagibacter isourolithinifaciens*, and also presented a higher abundance of some pro-inflammatory microbial including *Methanobrevibacter*, *Parvimonas*, Gammaproteobacteria and *Methanosphaera* compared with UM-A and UM-0.

These metabotypes have been reported to be differentially modulated upon consumption of different diets, for example, after the ingestion of walnuts for just 3 days [37]. After nut consumption, the genera *Bifidobacterium*, *Blautia*, and some microbes of the Coriobacteriia class, including the genus *Gordonibacter*, significantly increased exclusively in UM-B. In contrast, UM-A was less sensitive to walnut consumption, and some members of the Lachnospiraceae family decreased only in UM-A individuals. In the same context, a very recent human trial (MaPLE trial) has highlighted the usefulness of Urolithins metabotypes for assessing the effects of a polyphenol-rich dietary intervention (based on a polyphenol rich vs. a control diet) on intestinal permeability [77].

Concerning the association between these metabotypes and health, the current evidence suggests that equol and(or) ODMA producers may have a lower risk than non-producers, as reviewed by Frankenfeld [32]. Along the same line, and although several improvements in cardiometabolic biomarkers and, particularly, protection against menopausal symptoms have been described for equol (and, therefore, for equol producers), the underlying mechanisms are still unknown. Similarly, a higher cardiometabolic risk has been observed in overweight-obese UM-B individuals vs. UM-A and UM-0 and, specifically, UM-A can exert anti-inflammatory and anti-obesity activities, preserve the intestinal barrier, modulate the gut microbiota, and protect from oxidative stress, as demonstrated in several animal and in vitro models. However, more human studies are needed to confirm such results [54]. Whether these effects are produced by the specific gut microbial community, by the gut microbiome-derived metabolites or by a synergistic or additive effect remains unexplored. In fact, the actual evidence points towards considering metabolites as biomarkers of specific human polyphenol-related metabotypes rather than bioactive metabolites with differential impact on human health [118]. However, this issue deserves further research.

Along with the aforementioned metabotypes, recently, two novel metabotypes associated with resveratrol metabolism have also been identified: lunularin (LUNU)-producers and LUNU non-producers [55]. This study identified, for the first time, the specific metabolites generated by each metabotype, but future research focused on their associated microbiota and impact on health is needed.

It is important to mention, that, although we have focused on metabotypes from a “qualitative” point of view, the so-called “high producers” and “low producers” of specific polyphenols-derived metabolites could also allow clustering individuals and could allow predictions about their response to a specific nutritional intervention, such as in the case of moderate red wine intake [81]. In fact, red wine consumption increases the presence of beneficial bacteria only in those patients clustered as «high metabolizers» of polyphenols (measured in faeces) [8]. However, this classification could be influenced by several factors and there is also an important limitation that comes from establishing the appropriate cut-off for such clustering, which could be biased by the metabolite quantification technology used along with other factors.

To sum up, the characterization of gut microbiota associated with polyphenol-related metabotypes (composition and functionality through metatranscriptomics, metaproteomics and metabolomics approaches) is mandatory and should be included as a new variable to consider together with other personal, dense and dynamic data [97] in all personalized nutrition programs.

## Innovative formulations to improve the bioavailability of phenolic compounds

Phenolic compounds have gained significant attention due to their potential health benefits in managing the metabolic syndrome and associated pathologies. However, the low bioavailability of these compounds due to their high hydrophilicity is challenge in harnessing their therapeutic potential. For this reason, new innovative formulations that can enhance the bioavailability of phenolic compounds are being studied, improving their efficacy in the treatment and prevention of metabolic disorders. There are several factors that contribute to the low bioavailability of phenolic compounds, including their chemical structure, molecular weight, hydrophobicity, and instability in the gastrointestinal tract. These factors limit their absorption, distribution, and metabolism, resulting in reduced bioavailability and limited therapeutic effects. The chemical structure of phenolic compounds affects their solubility and stability. For instance, compounds with higher molecular weight or complex structures tend to exhibit lower bioavailability due to decreased absorption in the gastrointestinal tract. Additionally, the hydrophobic nature of phenolic compounds further hinders their solubility and subsequent absorption. Furthermore, the instability of phenolic compounds in the gastrointestinal tract, including degradation by digestive enzymes and the acidic pH, reduces their bioavailability. Rapid metabolism and elimination also contribute to their low systemic exposure and limited therapeutic effects.

Some innovative formulations to enhance bioavailability are nanoemulsions, nanoencapsulation and microencapsulation, liposomal delivery, solid lipid nanoparticles, cyclodextrin complexation or polymeric nanoparticles [10, 24, 127]. In this regard, encapsulation systems with proteins, polysaccharides, or lipids have been used to protect polyphenols from external factors and improve their bioavailability [43, 125]. One formulation to improve the bioavailability of polyphenolic extracts is their association with macromolecules derived from food, such as proteins. In this sense, proteins like β-lactoglobulin (β-Lg) have been used. This protein, heat-denatured to form nanoparticles (< 50 nm), has been associated with EGCG [112]. This association reduces the oxidative degradation of EGCG. Nanoencapsulation improved the organoleptic characteristics of EGCG, reducing its bitterness and astringency. Furthermore, β-Lg-EGCG nanoparticles withstood simulated gastric digestion, suggesting that they could serve as a natural vehicle for sustained release of EGCG. Nanoparticles of β- Lg and curcumin have also been studied [117]. These β-Lg-curcumin nanoparticles demonstrated rapid disintegration and nutraceutical release in simulated gastric fluid at pH 2, despite BLG’s known resistance to pepsin. However, they maintained their integrity in simulated gastric fluid at pH 5, followed by extensive degradation in simulated intestinal fluid, indicating the controlled release property of curcumin from the BLG nanoparticles when administered orally. Curcumin has also been studied in conjunction with the protein β-casein. This association forms a nanocomplex primarily driven by electrostatic interaction, which could enhance the bioavailability of curcumin [135]. Another study evaluated an ethanolic extract of *Artemisia dracunculus* L. complexed to soy protein [107]. This polyphenol-rich plant extract contained 2’,4’-dihydroxy-4-methoxydihydrochalcone. The complex formed with soy protein matrix improved insulin resistance both in vitro and in vivo. Furthermore, it enhanced the bioaccessibility, bioavailability, and efficacy of *A. dracunculus* polyphenols. This improvement was observed in both the TNO-1 human upper gastrointestinal tract model and C57BL6/J mice [107].

Other formulations to enhance the bioavailability of polyphenols involve their association with polysaccharides. Studies suggest that the adsorption of polyphenols to cellulose in plant cell walls, specifically dietary fiber, can readily occur in real food systems, influencing the bioavailability of polyphenols. One study indicated that the binding to cellulose was similar for polyphenols such as ferulic acid, gallic acid, catechin, and cyanidin-3-glucoside [92]. Chitosan, another positively charged polysaccharide, has been utilized to synthesize various polyphenol-chitosan conjugates due to its low toxicity, excellent biodegradability, and high biocompatibility, thereby enhancing their adhesion to the gastrointestinal mucosa. These conjugates have been prepared using different polyphenols, including gallic acid, caffeic acid, ferulic acid, salicylic acid, catechin, and EGCG [53]. Among these potential associations, few studies have examined their effects on metabolic syndrome. A study showed that the salicylic acid-chitosan conjugated exhibited in vitro activity as an antiplatelet adhesion and aggregation agent [57]. Another study assessed the preparation of flower-like resveratrol-loaded selenium nanoparticles/chitosan nanoparticles in mice with Alzheimer’s disease [132]. The intestinal microbiota plays a vital role in metabolic syndrome and cognitive impairment. These nanoparticles aided in restoring the intestinal microbiota and reduced lipopolysaccharide (LPS) formation and LPS-induced neuroinflammation. Various concentrations of endogenous bacteria, including Firmicutes and Bacteroidetes, were affected, aiding in the prevention of lipid deposition and insulin resistance. Therefore, the potential mechanism of action of this nanoparticle resveratrol-chitosan complex involves alleviating glycolipid metabolism, oxidative stress, neuroinflammation, and disruption of the intestinal microbiota [132]. The potential neuroprotective benefits of polyphenols in metabolic syndrome were also assessed utilizing microencapsulated mulberry fruit extract (*Morus alba* L.). This was conducted using a female Wistar rat model with induced menopause and metabolic syndrome [60]. Microencapsulation was carried out using maltodextrin dextrose equivalent 10 as the matrix, at a 9:1 (w/w) ratio with the mulberry extract. The characterized extract was abundant in polyphenols such as cyanidin-3-glucoside, gallic acid, and quercetin-3-*O*-rutinoside. The microencapsulated mlberry extract reduced memory impairment, oxidative stress levels, and AChE activity, while enhancing neuronal density and Erk phosphorylation in the hippocampus [60]. Another olive aqueous extract rich in polyphenols was microencapsulated using maltodextrins and dried by spray drying [90]. This formulation enables the protection of polyphenols from oxidation and heat, while also masking their bitter taste. This product improved the glucose and lipid profiles in both rats and humans. High-fat-fed rats treated with microencapsulated olive polyphenolic extract showed increased total plasma antioxidant capacity, elevated levels of HDL cholesterol, reduced LDL levels, and regulated insulin levels compared to the control group. Additionally, the human study involving healthy volunteers who consumed this microencapsulated product for 4 weeks showed lower levels of glucose, insulin, total cholesterol, and LDL [90].

Encapsulation is also a promising strategy for the polyphenols found in sea buckthorn leaves (*Hippophae rhamnoides* L.) [73]. Encapsulation was carried out using electrohydrodynamic techniques, which resulted in Zein microcapsules, gel microcapsules, and zein-gel microcapsules. These encapsulation methods provided a protective effect for the polyphenols against pH changes and enzymatic effects during digestion, thereby increasing their bioaccessibility. Furthermore, compared to the free extract, the encapsulation improved antioxidant activity and inhibition of enzymes related to metabolic syndrome such as α-glucosidase, α-amylase, and pancreatic lipase [73].

Finally, another very common model of polyphenol encapsulation is through association with lipids [10]. Some of the polyphenols encapsulated with lipids are quercetin, curcumin, or baicalein, encapsulated with lipids such as glyceryl monostearate, tripalmitin, gelucire, precirol, and miglyol, and surfactants such as Tween 80, polyethylene glycol, poloxamer, and lutrol F68 [65, 98, 121]. These nanoparticles improved absorption and distribution, enhancing the bioavailability of polyphenols. However, none of them have been tested in metabolic syndrome.

In addition to using proteins, polysaccharides, or lipids to encapsulate polyphenols, the incorporation into polymeric nanoparticles has also been studied [127]. One of these strategies for encapsulating polyphenolic compounds is the use of phyto-phospholipid complexes known as phytosomes [72]. Some of the polyphenols encapsulated in phytosomes include EGCG, quercetin, hesperidin, curcumin, luteolin, kaempferol, and rutin. These lipid-based nanoparticles, in addition to protecting polyphenols from external factors, enhance the solubility of lipophilic polyphenols in the aqueous phase and the membrane permeability of hydrophilic ones from the aqueous phase.

Cyclodextrins have also shown interesting applications in relation to the delivery of natural products and polyphenols for therapeutic, cosmetic and food applications [16]. These starch-derived natural polymers are able to create an interior hydrophobic cavity and a hydrophilic exterior layer. It is also interesting that olive pomace polyphenols like tyrosol, and hydroxytyrosol significantly increase their bioavailability when formulated in the presence of cyclodextrins [103]. Although these studies are performed using in vitro simulations, it is easy to elucidate the potential of these formulations human use as tyrosol and hydroxityrosol are in closely related to the benefits of olive products in the prevention of hypertension and associated disorders of the metabolic syndrome.

The development of innovative formulations offers promising strategies to improve the bioavailability of phenolic compounds for the management of metabolic syndrome. Nanoemulsions, nanoencapsulation, cyclodextrin complexation, and others have demonstrated their potential in enhancing the stability, solubility, and absorption of phenolic compounds, thereby increasing their therapeutic efficacy. However, further research is required to optimize these formulations, establish their safety profiles, and evaluate their long-term effects in clinical settings od metabolic syndrome. Overall, innovative formulations provide a platform for harnessing the full therapeutic potential of phenolic compounds in combating metabolic syndrome.

## Polyphenols in sports nutrition interventions: post-exercise recovery

Active people as well as trained athletes are the most important consumers of dietary supplements. The rationale of supplementation is that a regular diet does not provide sufficient nutrients for optimal performance. This concept was a main debate topic in the middle of the XX century after World War II. In this context, vitamins and minerals (particularly vitamin C, iron and calcium) were the most popular supplements consumed by athletes, following a common tendency in the postwar society. This assumption changed at the end of the XX century, and the reported motives argued that consuming supplements was related to disease prevention and maintaining an optimal health status [91]. Nevertheless, the paradigm changed at the end of the XX century, as documented during the Beijing Olympic Games [61]. New supplements followed traditional Chinese medicinal practices and based on chemical compounds present in vegetal sources but not considered nutrients. Polyphenols are in this category of supplements and are consumed for optimal recovery and performance. The interest of the scientific community in this topic increased in parallel with the interest of food companies to develop polyphenols as an attractive and productive business area. However, commercial polyphenols must be tested following scientific criteria to claim the benefits indicated in the marketing material. Moreover, consumers need to know the real properties of the different polyphenols and the method, dose, and moment for consumption [11]. Therefore, the idea of this section is to highlight key points taking into account the research in this scientific field and points of consideration in intervention protocols to obtain consistent and reproducible results. Recognizing the limitations that have led to deficiencies in intervention trials, as well as the extent of oxidative stress in the sports process that induces the recruitment of antioxidant defenses, may provide rational arguments for an optimal pharmacological approach.

Polyphenols display robust antioxidant and anti-inflammatory properties and are instrumental in postexercise recovery [78]. In this context, optimal recovery is essential for correct subsequent performance [106]. Intense physical activity requires more oxygen consumption and thereby mitochondrial activity. Since ATP production is not efficient at 100%, reactive oxygen species (ROS) appear as collateral products, causing fatigue and tiredness [96]. In addition, exercise-induced muscle damage during sustained efforts triggers the neutrophil phagocytic phase that increases ROS and pro-inflammatory cytokine production, causing muscle pain and soreness [50]. Nevertheless, ROS produced during exercise act as intracellular messengers in the activation of genes that code for antioxidant enzymes, including catalase, superoxide dismutase and glutathione-dependent enzymes [83]. However, excess ROS production due to intense exercise cannot be mitigated by endogenous antioxidants. In these cases, the intake of polyphenolic supplements can minimize ROS production, allowing for optimal recovery [35]. Recovery implies less oxidative damage, tissue repair and low inflammation.

In this context, the points to take into account in intervention protocols for future research must consider the following aspects:

- Administration form: polyphenols can be taken in different forms such as nutraceuticals (raw, purified or extracts), drinks and infusions. Polyphenol-rich foods are another way for consumption, as our research group showed with pomegranate juice during the recovery of marathon runners [35]. However, in these particular cases, the polyphenol content differs between the part of the plant (seeds, fruits and leaves) and the agro-ecological conditions of plant cultivation. In addition, the antioxidant action of a particular compound can be identified and compared to that of other compounds. For instance, in our research, verbascoside acid in *Lippia citriodora* extracts was identified as an activator of the antioxidant enzyme glutathione peroxidase [13]. In fact, when polyphenols are effective, they seem to act through antioxidant enzyme induction mediated by oxidative activation of Nrf2 and myeloperoxidase release [14, 31, 95]. In this case, a plant-enriched extract with a particular polyphenol can be an alternative to provide supplementation to athletes. In addition, the synergistic effects exerted between the different compounds present in the extract must be also considered.
- Intervention design: protocols must be designed according to the objective of the intervention, including cohort, experimental/control or double-blind studies, among others. Nevertheless, several points need to be considered, such as the time for supplement consumption that conditions the bioavailability and the mechanism of action in the organism [129]. To verify that polyphenols exert significant effects, reference values obtained from a representative population that does not consume the supplement (placebo or basal values from the beginning of intervention) are necessary. In this last context, the groups of participants (i.e., placebo and experimental) need to be homogeneous in terms of body composition, age, gender, diet consumed and athletic performance [83]. Exercise type is another key factor. Resistance exercises are related to more inflammation derived from exercise-induced muscle damage than endurance disciplines that produce high oxidative stress resulting from intense mitochondrial activity due to high oxygen consumption [17]. Exercise intensity is another variable to consider. The moment of the season (training, competition or precompetition) will impact the intensity when working in interventions with professional athletes [83]. In this context, eccentric exercises cause more muscle damage than concentric exercises [17]. In addition, recreational athletes do not follow rigid training schemes and for this reason, a homogenization period is mandatory, mainly if participants are sedentary and perform physical activity only during the intervention.
- Polyphenol dose: bioavailability is the first point to consider in this context [26]. Once absorbed, the administered doses do not have to interfere with the adaptation process of intracellular antioxidant defenses. As mentioned before, postexercise generated ROS can act as messengers to activate antioxidant-coding genes. Polyphenols should be administered in a dose and at a time that do not interfere with gene expression, allowing for accurate antioxidant adaptation. This process is known as exercise-induced hormesis: low or controlled stress favors adaptation, but high or uncontrolled stress causes damage [95].
- Markers of oxidative stress and muscle damage: the most commonly used markers to determine oxidative stress are malondialdehyde, TBARS (thiobarbituric acid-reactive substances) and isoprostanes for lipid peroxidation; and protein carbonyls for protein oxidation. More general markers include ORAC (oxygen radical absorbance capacity), TEAC (Trolox-equivalent antioxidant capacity) and TAS (total antioxidant capacity), among others [83]. Inflammation is evidenced by the presence of muscle proteins in circulation, accompanied by the presence of the inflammatory marker myeloperoxidase [12]. However, the levels of these proteins are conditioned by their half-lives in circulation, ranging from 1 day for creatine kinase to 7 days for lactate dehydrogenase [78]. This point must be considered to correctly interpret the obtained results. In this context, the time for blood sampling as well as the pharmacokinetics of the supplement are additional aspects to consider. Noninvasive and easy-to-handle technologies can allow for data collection at the same moment that the exercise is executed. References regarding this key aspect are scarce at present [33].

In conclusion, the potential of polyphenols in postexercise recovery is still of research interest. The molecular effects in biological systems as well as their interactions when administered as complex mixtures are questions that remain to be elucidated. Regarding performance, the reduction of oxidative stress and inflammation can enhance recovery times and overall endurance, allowing athletes to perform at higher levels and maintain their peak condition for longer periods. Additionally, polyphenols have been linked to improved vascular function and increased blood flow, which are crucial for optimal athletic performance. Recent research suggests that specific polyphenols, such as those found in green tea and certain berries, can boost nitric oxide production in the body, leading to better oxygen delivery to muscles during exercise. This can result in enhanced performance and reduced fatigue [51]. Altogether, the interest in natural and effective performance enhancers grows. Polyphenols are becoming a focal point for sports nutrition scientists and athletes alike, aiming to harness their benefits for improved training outcomes and competition goals. However, this is a point that anti-doping agencies and sports federations will need to consider in the future when developing regulations to ensure the health of athletes and maintain fair and balanced competitions.

## Corolary

This article provides constructive inspirations for advancing in the research of the applications, risk assessment, and metabolic effects of dietary polyphenols in humans. This is an innovative field of research with a clear application in improving human health and contributing to the so-called personalized nutrition. Undoubtedly, new results in the topics presented in the present review will be obtained in the coming years that will help to boost the field of metabolic implications, toxicology, and bioavailability of polyphenols.

## Data Availability

No datasets were generated or analysed during the current study.

## Declarations

### Clinical trial number

NA.
